# The amount of light reaching the leaves in seagrass (*Zostera marina*) meadows

**DOI:** 10.1371/journal.pone.0257586

**Published:** 2021-09-21

**Authors:** Mats Björk, Maria E. Asplund, Diana Deyanova, Martin Gullström

**Affiliations:** 1 Department of Ecology, Environment and Plant Sciences, Stockholm University, Stockholm, Sweden; 2 Department of Biological and Environmental Sciences, University of Gothenburg, Gothenburg, Kristineberg, Fiskebäckskil, Sweden; 3 School of Natural Sciences, Technology and Environmental Studies, Södertörn University, Huddinge, Sweden; Università della Calabria, ITALY

## Abstract

Seagrass meadows, and other submerged vegetated habitats, support a wide range of essential ecological services, but the true extents of these services are in many ways still not quantified. One important tool needed to assess and model many of these services is accurate estimations of the systems´ primary productivity. Such productivity estimations require an understanding of the underwater light field, especially regarding the amount of light that actually reaches the plants’ photosynthetic tissue. In this study, we tested a simple practical approach to estimate leaf light exposure, relative to incoming light at the canopy, by attaching light sensitive film at different positions on leaves of *Zostera marina*, eelgrass, in four seagrass meadows composed of different shoot density and at two different depths. We found that the light reaching the leaves decreased linearly down through the canopy. While the upper parts of the leaves received approximately the same level of light (photosynthetic photon flux density, PPFD) as recorded with a PAR meter at the canopy top, the average light that the seagrass leaves were exposed to varied between 40 and 60% of the light on top of the canopy, with an overall average of 48%. We recommend that actual light interception is measured when assessing or modelling light depending processes in submerged vegetation, but if this is not achievable a rough estimation for vegetation similar to *Z*. *marina* would be to use a correction factor of 0.5 to compensate for the reduced light due to leaf orientation and internal shading.

## Introduction

While it is well established that seagrasses benefit human needs through a wide range of ecosystem services, there is a need to understand the strength and variability of these services [1]. Underlying all ecosystem services and fundamentally building the biomass, the photosynthetic productivity supports growth and thereby removes nutrients from the surrounding water [2], and turns seagrass meadows into important carbon sinks [3]. As primary productivity is the storage of light energy in biomass, the estimation and modelling of productivity in submerged vegetation systems is dependent on an accurate estimation of the light absorption by plant canopies [4–6], especially when using non-intrusive methods [e.g. 7]. In aquatic systems, the light reaching the surface of a leaf is dependent on a range of factors, one being the orientation of the leaf surface relative to the incoming light [8], while also fluctuations in cloud cover, wave movement [9], water transparency and colour [10] may have large effects on photosynthesis of benthic algae and seagrasses [11]. Within the canopy, both steeper leaf angles and increased self‐shading decrease total light interception [8, 12]. This makes the actual light interception by leaves difficult to accurately estimate with a planar sensor, which is commonly applied at the top of the plant canopy [13]. However, a practical approach to measure the actual light interception by leaves in a canopy has been used on terrestrial plants by attaching a light sensitive colour acetate film with azo dyes directly to the surface of the leaves, and thereby including any changes in shading and light interception angles in the measurements [14]. This method has been used successfully to study competition for light between plant individuals of *Xanthium canadense* [15], assess the light regime in understory vegetation of forests [14, 16], and estimating light interception in cultured orchids [17], maize [18] and tomato [19]. Such light sensitive film has also been used to estimate the light interception of a commercial moss grown in water tanks [20], but has as far as we know not previously been used to study light interception in the natural marine coastal environment.

*Zostera marina* is an important habitat building species in temperate coastal areas of the Northern hemisphere and the most common rooted marine vegetation at the Swedish west coast, where it forms dense, highly productive communities [21, 22]. Here, we aimed to measure the actual levels of photosynthetic photon flux density (PPFD) intercepted at the seagrass leaf surface within *Zostera marina* meadows, and to explore if a general conversion factor for light interception by leaves relative to light measured at the top of the canopy can be calculated. We did that by attaching photosensitive film at different parts of the leaves of *Z*. *marina* in seagrass meadows and calibrating the light interception of these films with a PAR sensor. This research aims to supply a new simple tool to assess actual light interception to more accurately estimate and model productivity within seagrass meadows.

## Material and methods

### Study sites

The study was conducted in seagrass meadows dominated by eelgrass, *Zostera marina* (Linnaeus), in the vicinity of Kristineberg Marine Research Station in Fiskebäckskil at the Swedish west coast. In situ exposure of light sensitive film strips to ambient light conditions were performed in August, September and October 2020 in two sites–Kristineberg and Getevik, and at two depths (approximately 1 and 4 m), using SCUBA diving. The Kristineberg site is located in an open bay facing northeast and exposed to predominantly easterly winds. The sediment is sandy down to about 1.5 m depth, while below 1.5 m it becomes siltier and muddier with depth. The seagrass shoots in the shallow part (1 m depth) are shorter, thinner and grow denser than in the deeper parts of the meadow (2–6 m depth). Getevik is located in a sheltered narrow bay, which is open to the south and protected from the predominant winds. The sediments are very fine and muddy at all depths of the meadow. In Getevik, the seagrass shoots are more similar in size and density at different depths than in Kristineberg. At the shallow part in Getevik, *Z*. *marina* grows intermixed with *Ruppia maritima*. At both sites, there was a homogenous (relatively low) cover of filamentous algae typical for the region (mostly different species of the Ectocarpales), growing intermingled with seagrass shoots [23]. At the deeper sites, the cover of filamentous algae was negligible.

### Measurements and equipment

**Fig 1:**
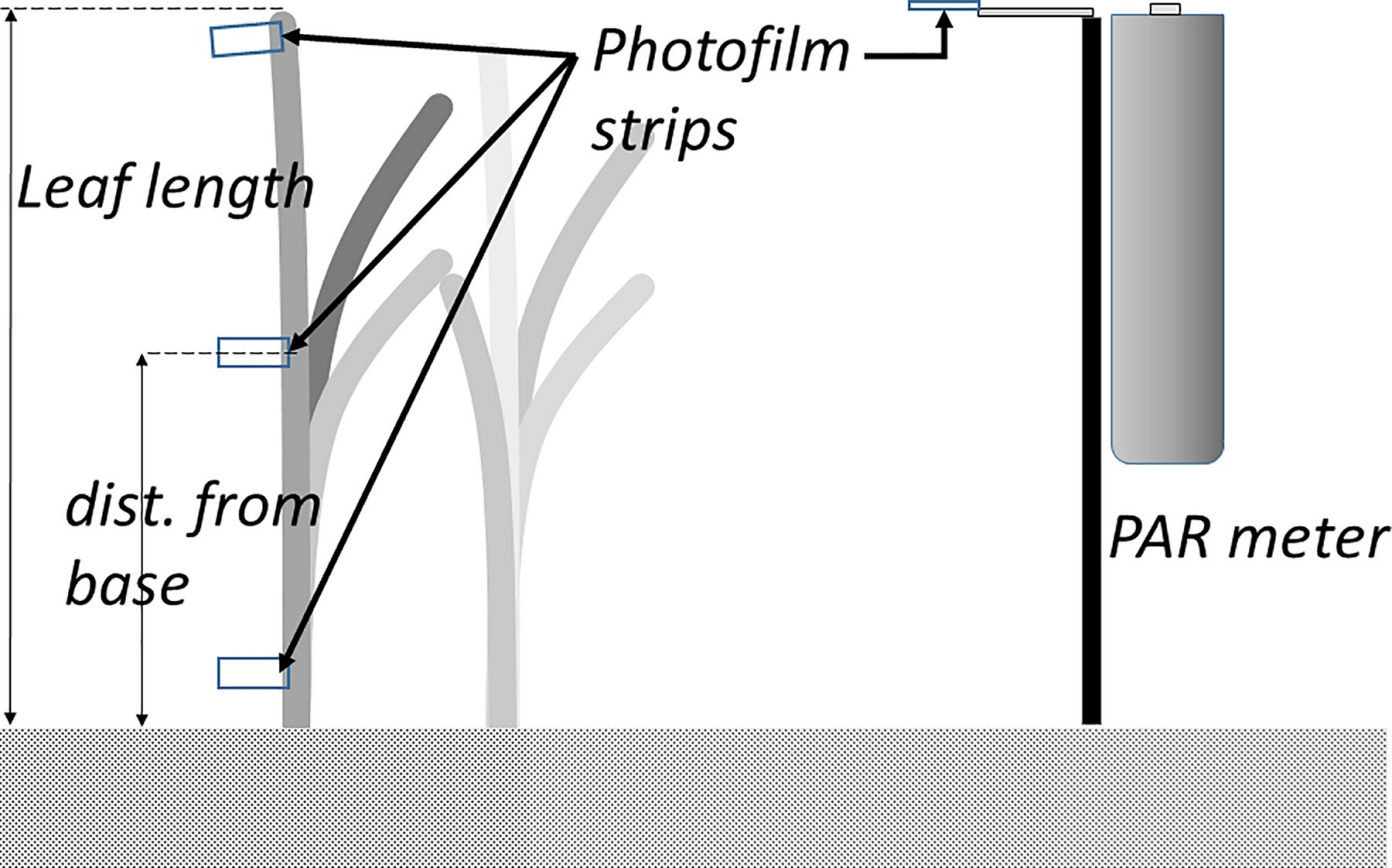
Experimental setup. The photo-filmstrips were attached along the seagrass leaf extending out from the leaf but at the same plane as the leaf surface. On a board attached to the PAR meter, separate filmstrips for calibration were attached horizontally in level with the meter.

To estimate light interception at different positions on seagrass leaves, we used a light sensitive colour acetate film impregnated with azo dyes (OptoLeaf O1-D, Taisei Fine Chemical Co., Ltd. Tokyo) that fades as a function of the amount of light absorbed [14]. The film has been shown to accurately reflect integrated PPFD in a variety of terrestrial habitats [14–19], including light with different spectral distribution [24]. The degree of fading of filmstrips was estimated by comparing the absorbance (at 492 nm) before and after exposure using a stationary spectrophotometer (Lambda 25, UV/VIS, Perkin Elmer, Waltham, MA, USA) at Kristineberg research station. The PPFD was estimated by PAR loggers (Odyssey, New Zealand) set to log at 10-second intervals, and positioned to measure incoming light at the top of the seagrass canopy (as indicated in Fig 1).

### Initial calibrations of filmstrips in situ (not including seagrass leaves)

Before starting the field exposures on seagrass leaves and to assure the applicability of this photo-film for estimating PPFD in the marine environment, a calibration of the method to the ambient marine conditions of our sites was performed by placing a total of 16 filmstrips on a horizontal board attached to the PAR sensor, positioned as indicated in the right part of Fig 1 (no filmstrips were attached to leaves). The filmstrips were then collected after different time intervals, from 2 to 72 hours after the start, and their degree of fading analysed. In this way, we tested the suitability of the method to cold-water environments and calculated a suitable exposure time to ensure that the tapes were collected within the accurate fading ratio (30 to 90%) as given in the instructions from the producer (Taisei Fine Chemical Co., Ltd. Tokyo). Based on the field calibration, we chose 48 h as a suitable time for exposure on seagrass leaves. A separate calibration was then performed together with each field exposure to ensure accurate calibration at different temperatures and light conditions.

We followed the procedures for estimating light exposure given by the manufacturer in all aspects except one. The film has an “up” and a “down” side, and the recommendation by the manufacturer is to use the “up” side of the photo-film only, as this side is more sensitive to light. However, we planned to use the film in a seagrass canopy with constantly changing leaf angles, and thus we considered it best to place the film strips extending out from the side of the leaf, like a flag, thereby intercepting light from both sides (as indicated in Fig 1). We therefore tested the sensitivity of the two sides of the tape by placing a set of 6 tapes on the calibration board upwards, and 6 tapes downwards and expose the films for 48 h in situ. We found that the absorption for the “up” side was 95.9% (±2.6 SE) of the integrated light recorded by the PAR meter, and for the “down” side 93.5 (±7.1 SE). Thus, we concluded that for our purposes we could use the film strips exposed from both sides.

### Field exposures of films attached to seagrass leaves

**Fig 2:**
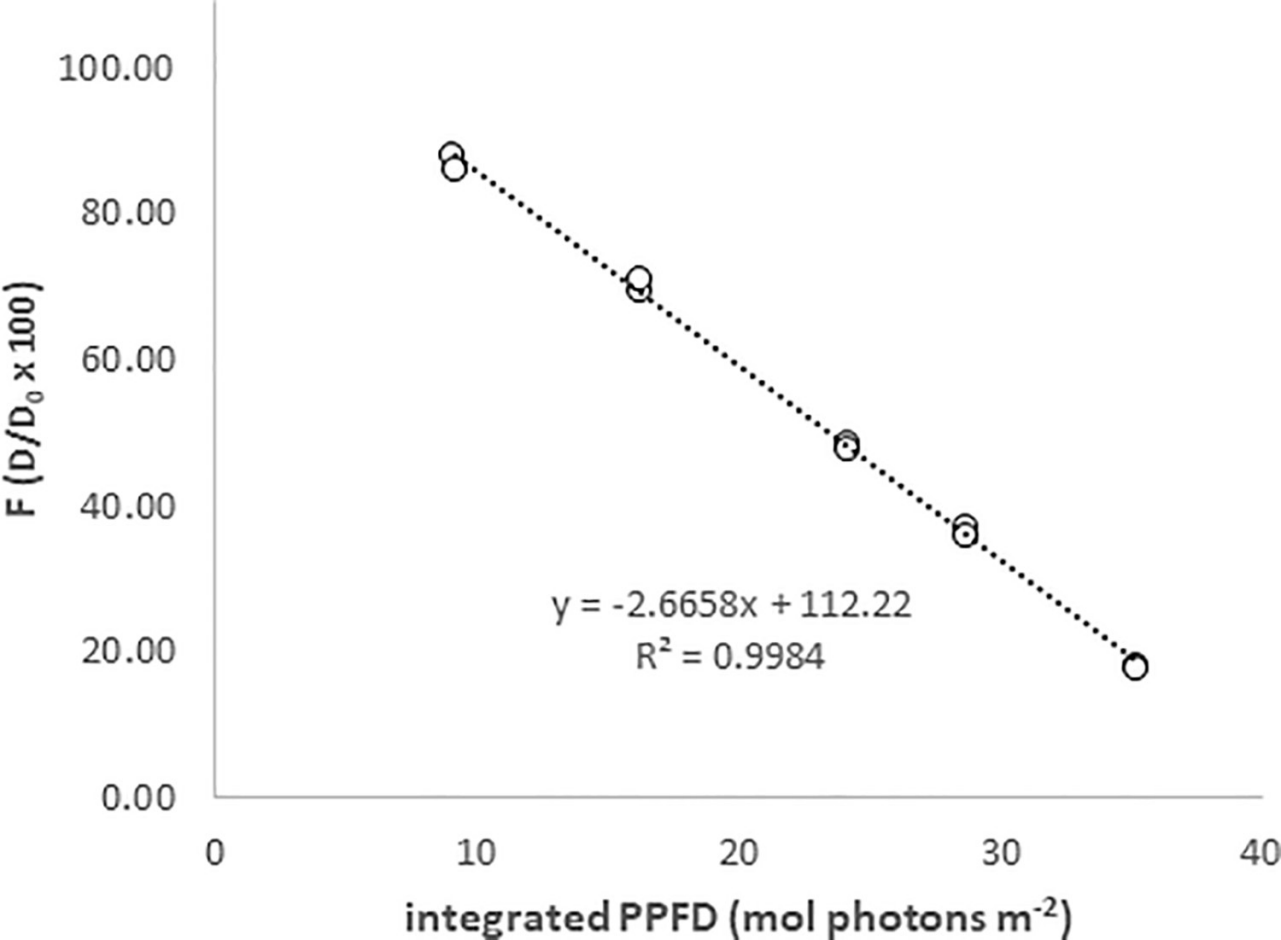
Example of an initial calibration curve, F vs. integrated PPFD, where film strips were placed at the same level and angle towards the surface as the PAR meter. Site: Kristineberg, depth: 1 m, temperature: 21°C.

For the light interception measurement, PPFD at the seagrass leaves was measured at different parts along the leaf using the light sensitive film. The integrated number of light quanta that reached the film was estimated by calibrating the film in the field with a PAR meter and producing a calibration curve (see text above and Fig 2). The film was cut in 1 cm broad sections and the filmstrips were attached (using a stapler) to ten randomly chosen seagrass leaves (of different shoots) at both depths in Kristineberg and Getevik (as illustrated in Fig 1). Filmstrips were attached at the bottom part, at the mid part and at the top part of leaves. At those filmstrips that were stapled to the top part of leaves, a “floater” made of a small piece of bubble wrap (about 2 x 2 cm) was attached in order to prevent the tip to sink due to the increased weight of the strips. In addition, PPFD at the top of the seagrass canopy was estimated by the PAR loggers. The total PPFD at the top of the canopy during the exposure period was calculated as the integral of the PPFD recorded by the sensor during the time the films were exposed to light in field.

When ending the exposure (after approximately 48h), the seagrass leaves with the attached filmstrips were cut right at the sediment surface and wrapped in a black plastic bag. The total length of each seagrass leaf and the distance from the base to where the filmstrips were attached were measured. At the research station, absorbance at 492 nm was measured and the fading ratio (F) was calculated from the absorbance of the film before use (D0) and after exposure (D) using the following formula (given in the manual; OptoLeaf O1-D, Taisei Fine Chemical Co., Ltd. Tokyo):

$$F=\frac{D}{D0}\;x\;100$$ (1)

The fading ratio, F, was then plotted against the integrated photosynthetic photon flux density (PPFD) measured by the PAR sensor for the time of the exposure period (Fig 2).

Additionally, the seagrass shoot density was estimated by randomly placing a 0.25 x 0.25 m quadrat (n = 4) at each site and at the different depths and counting the number of shoots within each sampling plot; the numbers were then recalculated as per square meter.

To calculate the percentage of canopy light that was intercepted at the different positions of the leaf (% PPFD_intercepted_), the light intercepted at the leaf surface (PPFD_intercepted_) was divided with the incoming light at top of the canopy (PPFD_canopy_):

$$\%\;PPFD\;intercepted=\frac{PPFD\;intercepted\;}{PPFD\;canopy}\;x\;100$$ (2)

The average PPFDs intercepted by leaves at each site and depth were calculated by plotting the %PPFD_intercepted_ against the distance from the base of the leaf to photofilm placement. The average %PPFD_intercepted_ was then calculated from the equation of the linear regression at a distance of half the average leaf length. To estimate a general average %PPFD_intercepted_ for all four sampling sites together, we plotted the %PPFD_intercepted_ against the relative distance from the base of leaf to photofilm placement according to:

$$Relative\;distance\;from\;base\;of\;leaf\;to\;photofilm=\frac{Distance\;from\;base\;of\;leaf\;to\;photofilm}{Total\;lengt\;of\;leaf\;}$$ (3)

Thus, a value of 1 is at the top of the leaf, and a value of 0.5 at the middle of the leaf.

### Data analysis

Simple linear regression analysis was applied to explore correlative relationships between fading ratio (F = D/D0 x 100) of the film strips and integrated light (PPFD) from the PAR sensor as well as between light intercepted by seagrass leaves (as a percent of the light on top of the canopy) and position along the leaves (measured from the sediment surface) for different sites separately and for all sites together using the IBM SPSS Statistics v. 27 software.

## Results

### Calibration of the method in situ

The correlation between the fading ratio F (= D/D0 x 100) of the film strips and integrated light (PPFD) from the PAR sensor was linear (R^2^ = 0.998, p < 0.001) within the recommended range of 30<F>90 (Fig 2). The exposure time for the filmstrips to fade within this interval was between 6 and 60 h, and we chose an exposure time for the leaves to be approximately 48 h. A separate calibration was then performed at each exposure.

### Seagrass light interception

**Fig 3:**
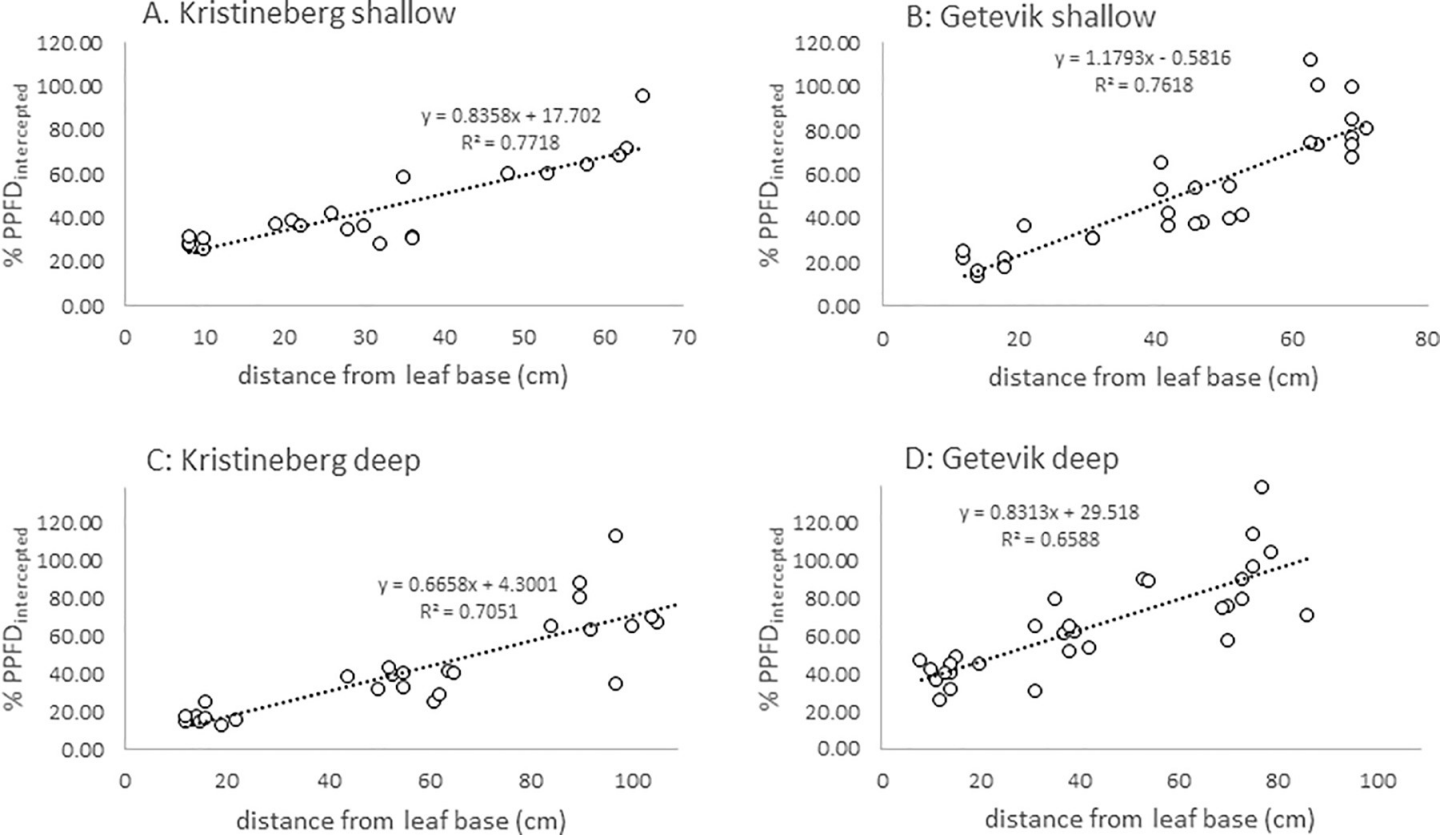
The amount of light intercepted by seagrass leaves as a percent of the light on top of the canopy (%PPFD_intercepted_) at different positions along the leaves (measured as distance in cm from the sediment surface). A-D are the light interception (at each of the four sub-sites) as a function of the actual distance from the base of the leaf.

At all four sampling sites, the light reaching the leaves decreased linearly from the top to the base of the leaves (Fig 3A–3D; R^2^ = 0.66–0.77, p < 0.001). The average %PPFD_intercepted_ ranged from about 40 to 60% of the light reaching the PAR sensor at the canopy surface (Table 1).

**Table 1:** Biometrics, depth, light at top of the canopy and the percentage of light that was intercepted at the leaf surface.

	Shoot density (shoots m-2, ±SE)	Average leaf length (cm, ±SE)	Depth (m)	integrated light at the top of the canopy (mol m^-2^d^-1^)	Light interception by leaves (% of canopy light)
**Kristineberg shallow**	364±21	60.7±1.6	0.7	2.12	43%
**Getevik shallow**	192±15	67.1±0.9	1.2	4.19	39%
**Kristineberg deep**	160±10	97.4±2.0	4.0	1.67	37%
**Getevik deep**	132±12	75.9±1.3	3.9	1.35	61%

**Fig 4:**
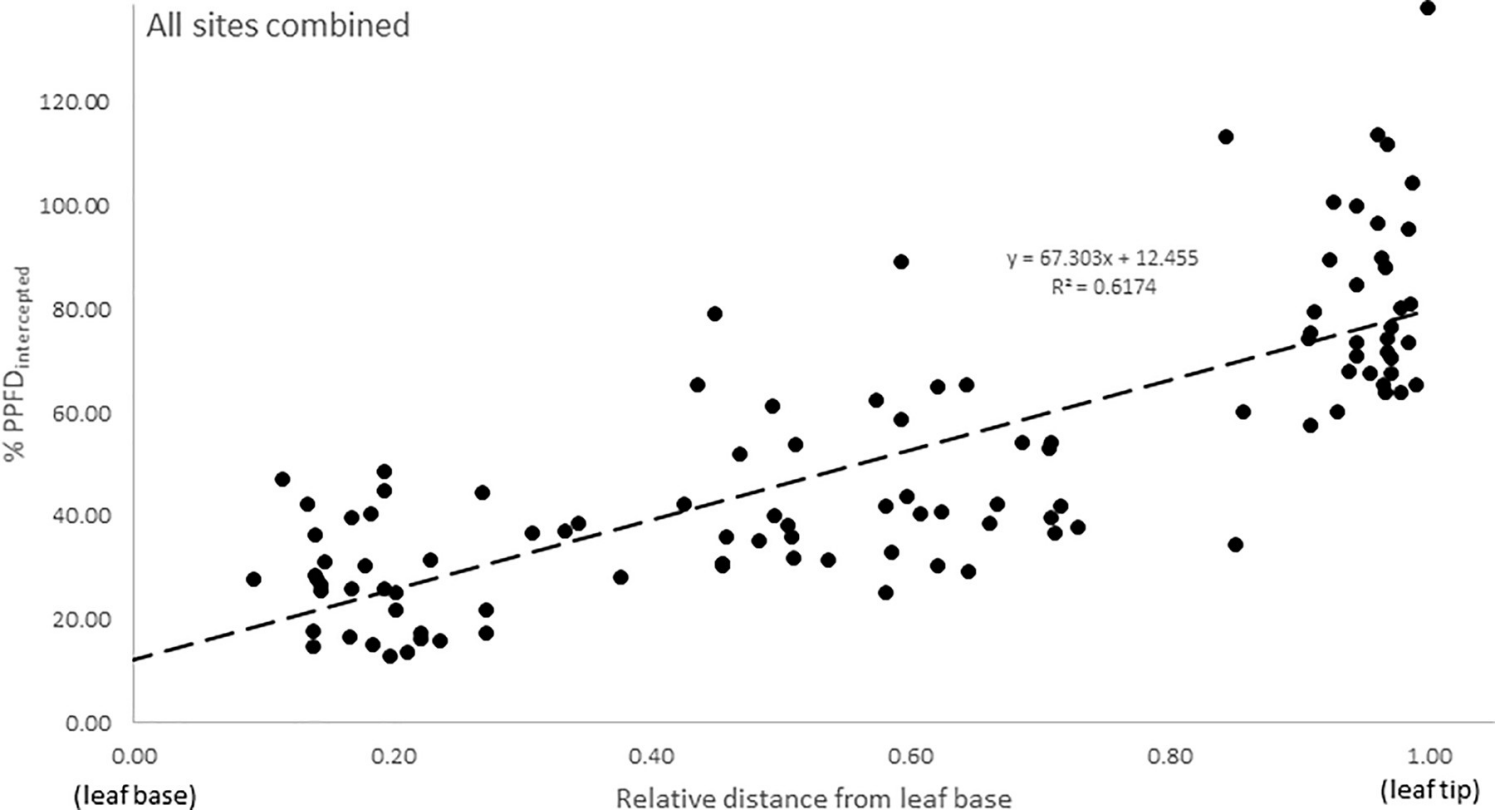
All sites combined together. The X-axis shows the amount of light intercepted by the leaf as the percent of the light on top of the canopy (%PPFD_intercepted_). The Y-axis shows the relative position along the leaf, where 1 is the top of the leaf and 0.5 is the mid part of the leaf.

By plotting PPFD_intercepted_ to the relative distance from leaf base (Eq 3), it was possible to estimate a general light exposure coefficient for the two meadows (Fig 4; R^2^ = 0.62, p < 0.001). This showed that overall, the total average PPFD reaching the full length of seagrass leaves was 48% of what the PAR meter at the canopy top was recording.

**Fig 5:**
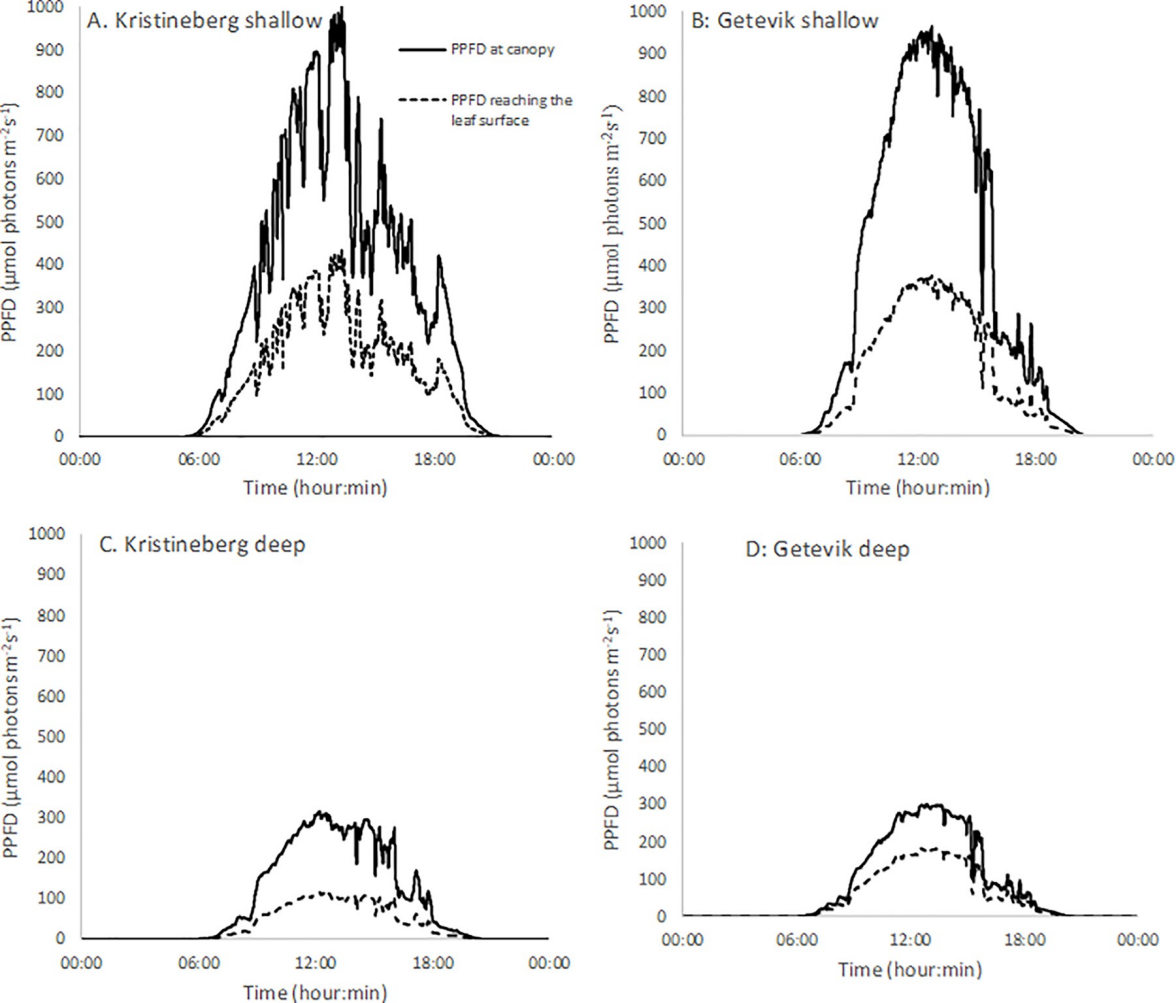
PPFD measured by the light loggers at the seagrass leaf canopy (solid line), and the estimated light interception at the seagrass leaves (PPFD_intercepted_) calculated from the average %PPFD_intercepted_ in Table 1 (broken line).

Fig 5 shows the measured light from the PAR meters, during 24 h, at the top of the canopies together with the estimated light interception by the leaves as calculated from the respective average light exposure values (Table 1). The PPFD on top of the canopies was similar for the shallow sites, reaching levels near to 1000 μmol photons m^-2^ s^-1^ at midday, and similarly the two deeper sites both reached close to 300 μmol photons m^-2^ s^-1^ at midday.

## Discussion

This study clearly showed that light measured on top of the seagrass canopy considerably overestimates the light that actually is intercepted by the leaves, and that such values can be corrected to better reflect the true light conditions within the seagrass meadow. Approximately half of the light reaching the top of the canopy was intercepted by the seagrass leaf surfaces. This was similar at both sites and the two depths, where the average %PPFD_intercepted_ ranged between 40 and 60%, even though shoot density and average leaf length differed between the different sites and depths. Since the leaf length was higher when the shoot density was lower, it is probable that self-shading were at similar levels in the four sub-sites. Overall, the data suggest that a general ratio between light at top of the canopy and the light interception by the leaves is close to 0.5, at least for plant species with a morphology similar to *Zostera marina*, such as *Cymodocea* spp. and *Thalassia* spp., and possibly also for the larger species *Enhalus acoroides* and *Posidonia oceanica*. For seagrasses with a completely different morphology, e.g. *Halophila* spp. and *Syringodium* spp., or species with a distinct top layer canopy such as *Thalassodendron ciliatum*, we do not recommend using this simple 0.5 correction factor. For plants with such morphologies, it is advisable to do a specific study for each species. Even though this study was performed on seagrass, we believe our method can also be applied on freshwater plants.

The amount of light needed to saturate photosynthesis in marine plants varies with environmental conditions and plants generally acclimate to prevailing light regimes to efficiently utilise available light [25]. For *Z*. *marina*, light saturation levels have been reported to range from about 100 μmol photons m^-2^ s^-1^ for in situ estimations in Massachusetts US [26] to 230 and 300 μmol photons m^-2^ s^-1^ for shallow growing seagrass in California, US [27] and at the shallow Kristineberg site [28], respectively. These levels correspond well with the compensated light regimes in our study (which ranged from c. 100 to 400 μmol photons m^-2^ s^-1^), whereas the uncompensated light regimes (measured on top of the seagrass canopy) were considerably higher (up to 1000 μmol photons m^-2^ s^-1^) (Fig 5). So, even though it is difficult to use literature values for parameters like light saturation, the plants we used in this study appear to be acclimated to light levels that are corresponding closely with our corrected light values (as in Fig 5).

It is possible that the orientation of the leaf surface, often close to vertical in seagrasses, can increase light capture when the sun is low, while decreasing light capture at noon when the sun is at its highest. In terrestrial plants, it is suggested that steeper leaf angles function to reduce exposure to excess light levels during the middle of the day [12]. In that way, the light interception will be more evenly distributed over the light period, resulting in a more effective use of light. Due to the limitation of our study, we were however not able to quantify this, and suggest it to be an interesting study for the future.

Overgrowth by ephemeral or epiphytic algae (which may vary substantially across seasons in temperate waters), or biofilms attached to the leaf surface would decrease the light penetration even further. It has been shown that different epiphytic algae can drastically decrease light penetration to the seagrass leaves [29]; in such cases, the correction factor suggested here (i.e. 0.5) must be supplemented with a measure of the light attenuation by the epiphytic load (e.g. following the methodology suggested in [29]). In the present study, no excess of epiphytic algae was observed, but at high densities the light reduction by algal overgrowth has been reported to be substantial [28–30] and should therefore be considered in productivity models. Another factor that changes while light is penetrating through algal overgrowth is the light quality. Such studies are however rare and while significant effects of light quality on growth, morphology, seed germination and survival have been shown in *Halophila ovalis* [31], a study on *Zostera marina* showed no inhibition of photosynthetic rates when light was shifted more towards the green part of the spectrum [28].

Both photosynthetic rates and respiration have been shown to vary along the leaves of *Z*. *marina* [32], which could possibly be connected to the differences in light interception. Also regulation of genes involved with photosynthesis (in *Posidonia oceanica*) are influenced by ambient light levels [33, 34] and in *Zostera muelleri* light levels has been shown to affect metabolic enzymes for photosynthesis and the metabolism of carbohydrates and amino acids [35]. Moreover, it has been shown that mitochondrial respiration in *Z*. *marina* are affected by light levels during daytime [36, 37]. With the light correction method presented here, it is possible to make more accurate predictions of light dependent processes within marine vegetated areas by calculating light interception at the different levels within the canopies.

We recommend that the actual light interception of photosynthetic tissue is measured when assessing or modelling light dependent processes in seagrass and other submerged vegetation. If this is not possible, a rough estimation of light interception would be obtained by using a correction factor of 0.5 to convert the light at the canopy to light exposure at the leaf surface for plants similar in morphology to *Z*. *marina* growing in densities within the same range as we studied here.
